# The plasmid-encoded lactose operon plays a vital role in the acid production rate of *Lacticaseibacillus casei* during milk beverage fermentation

**DOI:** 10.3389/fmicb.2022.1016904

**Published:** 2022-10-06

**Authors:** Xiaoxia Li, Zhengyuan Zhai, Yanling Hao, Ming Zhang, Caiyun Hou, Jingjing He, Shaoqi Shi, Zhi Zhao, Yue Sang, Fazheng Ren, Ran Wang

**Affiliations:** ^1^College of Food Science and Nutritional Engineering, China Agricultural University, Beijing, China; ^2^Department of Nutrition and Health, Key Laboratory of Functional Dairy, Co-constructed by Ministry of Education and Beijing Government, China Agricultural University, Beijing, China; ^3^School of Food and Health, Beijing Technology and Business University, Beijing, China

**Keywords:** *Lacticaseibacillus casei*, acid production rate, comparative genome, *lac* operon, fermented milk-beverage

## Abstract

*Lacticaseibacillus casei* is used extensively in the fermented milk-beverage industry as a starter culture. Acid production capacity during fermentation is the main criterion for evaluating starters although it is strain-dependent. In this study, the acid production rates of 114 *L. casei* strains were determined and then classified into high acid (HC), medium acid (MC), and low acid (LC) groups. Comparative genomics analysis found that the *lac* operon genes encoding the phosphoenolpyruvate-lactose phosphotransferase system (PTS^Lac^) were located on plasmids in the HC strains; however, it is notable that the corresponding operons were located on the chromosome in LC strains. Real-time PCR analysis showed that the copy numbers of lac operon genes in HC strains were between 3.1 and 9.3. To investigate the relationship between copy number and acid production rate, the *lac* operon cluster of the HC group was constitutively expressed in LC strains. The resulting copy numbers of *lac* operon genes were between 15.8 and 18.1; phospho-β-galactosidase activity increased by 1.68–1.99-fold; and the acid production rates increased by 1.24–1.40-fold, which enhanced the utilization rate of lactose from 17.5 to 42.6% in the recombinant strains. The markedly increased expression of *lac* operon genes increased lactose catabolism and thereby increased the acid production rate of *L. casei*.

## Introduction

Dairy protein-based beverages have received considerable research interest, because of the increasing consumption of health-promoting foods containing protein. The dairy beverages market is a very dynamic segment of the dairy industry, and the global dairy-based beverages market reached US$13.9 billion in 2021 (Abbas et al., 2022). Fermented milk beverages supplemented or enriched with functional ingredients such as bio-active peptides or probiotics occupy the largest segment within the dairy-based beverage market (Champagne et al., 2018; Fazilah et al., 2018). In addition to basic nutrition, fermented milk beverages offer health benefits to the consumer, e.g., prevention of digestive diseases, enhancement of immunity, and reduction of infection risk (Matsuzaki et al., 2004; Sanggaard et al., 2004; Shiby and Mishra, 2013). The requirements of the Chinese industry standard (NY1799-2004) for fermented milk beverages are based on probiotic microbial counts (>10^6^ CFU/ml) and acidity, which must be greater than 25°T. The main function of the starter in milk fermentations is to produce organic acids by fermenting sugars. As the major acid end product, lactic acid not only provides the required acidity for fermented milk drinks, but also provides their characteristic taste, through its content and proportion (Leongmorgenthaler et al., 1991). Thus, the acid production rate is an important characteristic of a starter, because slow acid production may have major effects on the quality of the final product and increase the costs of industrial lactic fermentation.

Fermented milk beverages containing probiotic strains are now well-established products in the global market; the fermentation is usually promoted by starter lactic acid bacteria (LAB) species such as *Lactobacillus*, *Streptococcus*, and *Bifidobacterium* (Fooks et al., 1999; Berhe et al., 2018). *Lacticaseibacillus casei* is an LAB that can be isolated from a variety of diverse habitats and has remarkable ecological adaptability, which contributes to the stability and persistence of acid production by *L. casei* during fermentation (Samet-Bali et al., 2010). *L. casei* has been isolated from dairy products, plant materials, as well as the human oral cavity and gastrointestinal tract (Cai et al., 2009). *L. casei* is mainly used in fermented milk beverages as a separate-starter, because of its efficient acid production; some examples of commercial fermented milk beverages contain strains of *L. casei* Shirota, *L. casei* Danone, and *L. casei* 01™ (Duar et al., 2017; Turkmen et al., 2019).

The acid production rate during *L. casei* fermentation is influenced by several factors:

### Type of carbohydrate

Almost all *L. casei* strains are able to ferment common hexose sugars, including glucose, fructose, mannose, and N-acetylglucosamine, as well as the disaccharides lactose, maltose, and cellobiose. The ability of *L. casei* to ferment other sugars is strain-dependent, their carbohydrate utilization being influenced by the environment they are isolated from (Ganzle and Follador, 2012).

### Hydrolytic capacity for disaccharides

As a facultatively hetero-fermentative species, the energy required for *L. casei* growth depends on the hydrolysis and metabolism of carbohydrates. Lactic acid production depends on glycoside hydrolase activity, which is highly variable (Hidalgo-Morales et al., 2005; Felis and Dellaglio, 2007). For example, the rapid hydrolysis of lactose implies the presence of a highly active β-galactosidase (EC 3.2.1.23) that enables *L. casei* ATCC334/64H to rapidly hydrolyze lactose into glucose and galactose-6-phosphate (Witt et al., 1993). The galactose-6-phosphate is metabolized to organic acids, *via* the tagatose-6-phosphate pathway, which is coded by the *galR-galKTEM* gene cluster (Tsai and Lin, 2006; Bidart et al., 2018). In addition, a trehalose 6-phosphohydrase (EC 3.2.1.93) of the GH13 family catalyzes the hydrolysis of trehalose, a β-glucosidase (EC 3.2.1.21) is the rate-limiting biocatalyst of cellobiose hydrolysis, and β-D-fucosidase (EC 3.2.1.38) is the rate-limiting biocatalyst for fucose hydrolysis (Ganzle and Follador, 2012; Buron-Moles et al., 2019). These glycoside hydrolases increase the ability of *L. casei* to hydrolyze various different carbohydrates and produce organic acids, to increase the acid production rate.

This study aimed to group *L. casei* isolates by performing phenotypic analysis of acid production rate and exploring the genetic basis of lactose metabolism by different strains. The association between acid production rate and the *lac* operon was established, which provides a theoretical basis for screening of starter strains for rapid acid production, for use in fermented milk beverages.

## Materials and methods

### Samples and *Lacticaseibacillus casei* isolation

The products from which the isolates came originated from 29 different regions in 12 provinces (Yunnan, Tibet, Guangxi, Gansu, Anhui, Shanxi, Guizhou, Guidong, Beijing, Chongqing, Xinjiang, and Inner Mongolia) in China between 2006 and 2020. Samples were derived from dairy products (fermented milk, koumiss, and qula), plant materials (pickles, wine), cured meat, and human isolates (vaginal, feces). Detailed information on samples and isolates is listed in Supplementary Table S1.

Suitable dilutions of sample isolates were inoculated in triplicate on De Man, Rogosa, and Sharpe (MRS) agar containing the pH indicator bromocresol purple (BCP; Sigma-Aldrich, St. Louis, MO) and cycloheximide (0.01% v/v) to prevent fungal growth. After incubation at 37°C for 48 h under anaerobic conditions, colonies with distinct morphological differences (color, shape, and size), which were producing acid, according to the BCP, were selected and purified by streaking on MRS agar (supplemented with 10 mg/l vancomycin). Representative colonies were selected (~10% of the observed count) and tested for positive Gram staining and the absence of catalase activity. The screened isolates were stored in cryoprotectant (12% w/v skim milk containing 10% glycerol) and cultured in MRS broth (Oxoid) at 37°C overnight before further experimentation. Microbiological analyses of the 114 isolates were conducted using methods including conventional phenotypic identification, 16S rRNA sequence, and species-specific PCR (Supplementary Table S2; Liu et al., 2012; Colombo et al., 2018, 2020).

### Milk fermentation

The milk fermentation medium was reconstituted skim milk powder (13% w/v; Fonterra™, Auckland, New Zealand) with sucrose (5% w/v) and glucose (3% w/v). The medium was two-stage (5ΜPa, 15 MPa) pressure-homogenized (Sfakianakis et al., 2015), sterilized by heating for 60 min at 95°C, inoculated with 5 × 10^6^ CFU g^−1^ of an *L. casei* isolate, and then incubated at 37°C for 96 h.

### Determination of titratable acidity and viable microbial counts

The titratable acidity of the fermented milk was determined according to the China National Standard GB 5009.239–2016 (Jia et al., 2016). Fermented milk (10 g) was added to phenolphthalein solution (2 ml, 1% w/v in ethanol) and the mixture was titrated with standardized 0.1 M NaOH. The titratable acidity was calculated and expressed as degrees Thorner (°T; Alferez et al., 2001).

Viable microbial counts. The viable microbial count of *L. casei* isolates in milk fermentations was determined by counting colonies on MRS agar as described previously (Sah et al., 2014). Plates were incubated for 36–48 h at 37°C. The number of viable cells per gram was counted and expressed as logCFU g^−1^. Correlations were evaluated by the testing significance of Pearson’s correlation coefficient (titratable acidity, bioactive compounds, pH) and Spearman correlation coefficient (growth).

### Acid production rate

We proposed a new method for the determination of acid production rate based on the titratable acidity and viable microbial count. The acid production rate of 114 isolates was calculated by dividing the acidity by the viable microbial count. Cluster analysis of *L. casei* strains was performed using IBM SPSS statistics 26.

### Preferred carbohydrate source determination

HC and LC strains were inoculated (5 × 10^6^ CFU g^−1^) in MRS broth containing a carbohydrate carbon source (0.5% w/v) and bromocresol purple (0.1% w/v). All incubations were performed at 37°C and in quadruplicate. The strain was considered to grow successfully on the tested sugar when the turbidity at OD_600_ was >0.8. The blank was un-inoculated MRS broth.

### Sugar content and composition of fermented milk samples

Carrez clarification reagents 1 and 2 were prepared by separately dissolving potassium hexacyanoferrate (10.60 g, Sigma) and zinc acetate dihydrate (21.90 g, Sigma) in water (100 ml). Fermented milk (5 g) was weighed accurately into graduated tubes (100 ml) and dissolved in water (25 g). Carrez reagents 1 and 2 (2.5 ml each) were added sequentially with magnetic stirring for 30 min, then the mixture was centrifuged at 5000 × *g* for 15 min. Extracts were made to 100 ml with water, filtered (discarding initial filtrate) and an aliquot passed through a 0.22 μm membrane filter (Indyk et al., 1996). A control milk powder sample was included in each sample set to monitor method performance.

Sugar content was measured by high-performance liquid chromatography (HPLC) on a Model 2,695 HPLC (Waters Corporation, Milford, MA), fitted with a Bio-Rad Aminex®HPX-87P column (300 mm × 7.8 mm × 9 μm; Bio-Rad, Hercules, CA) and a Model 2,414 differential refractive index detector (RID). The mobile phase was aqueous sulfuric acid (5 mM) at a flow rate of 550 μl/min, with the column temperature set at 60°C.

### Draft genome sequencing and annotation

Bacterial DNA isolation and purification. Overnight cultures with an OD_600_ of 0.8–1.0 were collected by centrifugation and washed with TES buffer (50 mM Tris-Cl; 30 mM EDTA; 25% Sucrose, pH 8.0). The cells were lysed by the addition of 100 μl of 50 mg/ml lysozyme solution in TE, with subsequent incubation at 37°C for 30 min. A total *L. casei* DNA extract was prepared as described previously (Pushnova et al., 2000). Cell lysates (50 mM Tris-Cl, pH8.0; 10 mM EDTA, pH 8.0; 50 mM sodium chloride; 60 mM sodium acetate, pH 5.2; 1% w/v SDS) were heated at 65°C for 30 min and extracted with phenol/chloroform.

DNA samples (1 μg) were cleaved into 400–500 bp fragments using a Covaris (Woburn, MA) M220 Focused Acoustic Shearer, following the manufacturer’s protocol. Illumina sequencing libraries were prepared from the sheared fragments using the NEXTflex™ Rapid DNA-Seq Kit (PerkinElmer Applied Genomics). Libraries then were used for paired-end Illumina sequencing (2 × 150 bp) on an Illumina NovaSeq 6,000 (San Diego, CA). Assembly of the clean reads was performed using SOAPdenovo2 (Koren et al., 2017). Glimmer (Delcher et al., 2007) was used for CDS prediction, tRNA-scan-SE (Borodovsky and Mcininch, 1993) was used for tRNA prediction, and Barrnap (Victorian Bioinformatics Consortium, Australia) was used for rRNA prediction. The predicted CDSs were annotated using the NR, Swiss-Prot, Pfam, GO, COG, and KEGG databases, using the sequence alignment tools BLASTP, DIAMOND, and HMMER. Each set of query proteins was aligned with the databases, and annotations of best-matched subjects (e-value <10^−5^) were obtained for gene annotation.

### Comparative genomics/orthology prediction

All protein sequences of the six exemplar *L. casei* genomes were subjected to an orthology prediction using OrthoMCL (Fischer et al., 2011), with thresholds of: E-Value: 1E-5, percent identity cutoff: 0, and Markov inflation index: 1.5. Comparative genomic analyses for COGs were performed using the similarity clustering program implemented in ERGO (Huerta-Cepas et al., 2016). The core genomes were mapped to the bacterial COGs and KEGG database to evaluate the main functional COG categories related to the genome (Huerta-Cepas et al., 2016). Screening of core genes (genes contained in all gene families) and unique genes (genes contained only in a specific gene family) was based on homologous gene analysis.

### Copy number determination

The relative copy number was assessed by the quantification method (Alvarez-Martin et al., 2007). The elongation factor Tu gene *tuf* (GenBank Accession No. AJ418937.2), identified as a chromosomally encoded single-copy gene, was used as the reference gene (Chavagnat et al., 2002). A 180 bp fragment of the *tuf* gene was amplified with primers tuf2-F and tuf2-R (Supplementary Table S2) and a 102 bp fragment of the *lacG* gene was amplified with the primers lacG-F and lacG-R (Supplementary Table S2). Real-time PCR was conducted with the following cycling conditions: 95°C for 5 min, followed by 40 cycles of 95°C for 20 s, 60°C for 30 s, and 68°C for 30 s each. The relative copy number was calculated using *N*_relative_ = (1 + E)^−△CT^ (Alvarez-Martin et al., 2007), where E and ΔC_T_ represent the PCR amplification efficiency and the difference between the threshold cycle number (C_T_) of the *tuf* and *lacG* reactions, respectively.

### Construction of expression vector

General molecular techniques, including DNA electrophoretic analysis, recovery, and storage, were performed using standard protocols. pSIP600 (Supplementary Figure S2) was pSIP502 vector without *nisRK* (Sorvig et al., 2003). Plasmid isolation from both *E. coli* and *L. casei* was performed using a Plasmid Kit according to the manufacturer’s instructions (OMEGA Bio-tek Inc., Doraville, GA). PCR was performed using Q5 high-fidelity DNA polymerase (New England Biolabs, Ipswich, MA). Primers used in PCR reactions were synthesized by Sangon Biotech (Beijing, China) and are listed in Supplementary Table S2. The purified PCR products were cloned directly into *Bgl*II-*Hind*III-digested pSIP600, using a ClonExpress Ultra one-step cloning kit (Vazyme Biotech, Beijing, China). The recombinant vector pSIP601 (Supplementary Figure S3) was transformed into *E. coli* DH5α using standard heat shock transformation (Watanabe et al., 1994) and introduced into LC strains by electroporation (1.5 kV, 400 Ω, 25 mF; Welker et al., 2015). Erythromycin was added as follows: 500 μg/ml for *E. coli* and 10 μg/ml for *L. casei* strains. *L. casei* LC_N31/pSIP601, LC_N80/pSIP601, and LC_N88/pSIP601 recombinant strains were generated under erythromycin resistance selection. DNA sequencing was performed by Sangon Biotech and the results were analyzed with DNAMAN software.

### 6-phospho-β-galactosidase assay

The 6-phospho-β-galactosidase (LacG) activity of selected strains was assayed as described previously (Hengstenberg et al., 1969; Bidart et al., 2018). A measured amount of the microbial sample (10^4^CFU) was dissolved in Tris-hydrochloride buffer (0.9 ml, 0.1 M, pH 7.6, containing 0.05 M NaCl and 0.05 M MgCl_2_). The reaction was started by adding *o*-nitrophenyl-β-D-galactopyranoside-6-phosphate (ONPG-6-P; 3 μmoles; Toronto Research Chemicals, Canada) in distilled water (0.1 ml) to the enzyme solution, then the mixture was incubated at 30°C. The assays were repeated twice and in triplicate and the increase in absorbance was monitored at 405 nm. One unit of LacG activity was defined as 1 nmol/h of ONP released at 30°C by 10^4^CFU of *L. casei*.

### Genetic analyzes

Sequence similarity was detected with BLAST (https://blast.ncbi.nlm.nih.gov/Blast.cgi) and multiple sequence alignments were performed with Clustal W (Thompson et al., 1994). Putative sugar and amino acid metabolic pathways were predicted by KEGG (http://-www.genome.jp). Carbohydrate-active enzymes were identified using the CAZy database (http://www.cazy.org; Cantarel et al., 2009). ANIb calculation (Richter and Rossello-Mora, 2009) was performed using pyani (v0.2.7; https://cloud.majorbio.com/) to evaluate distances between all *L. casei/paracasei* genomes. The presence of an N-terminal signal peptide sequence was predicted using SignalP 6.0 (https://services.healthtech.dtu.dk/service.php?SignalP-6.0; Petersen et al., 2011).

Nucleotide sequence accession numbers. The draft genome sequences of the six exemplar isolates were submitted to GenBank under the following accession numbers: LC_N16 (SAMN24532469), LC_N17 (AMN24532470), LC_N40 (SAMN24532471), LC_N31 (SAMN24532472), LC_N80 (SAMN24532473), and LC_N88 (SAMN24532474).

## Results

### Isolation and identification of *Lacticaseibacillus casei* strains

A total of 199 samples were collected from 29 geographical regions, in 12 Chinese provinces, for isolation and purification. Isolates were identified as LAB based on positive Gram staining assays, the absence of catalase and oxidase activity, and microscopic observation of cell morphology. A panel of 472 LAB isolates was obtained from the samples and LAB were chosen for taxonomical identification by sequencing of the PCR-amplified 16S rRNA; there were 171 strains of *L. casei/parcasei/rhamnosus* subspecies (Supplementary Table S1, set A; Liu et al., 2012). *L. casei*-related taxa strains were specifically identified with housekeeping loci (*pheS*, *recA*, *rpoC*, *tuf*, and *uvrC*; Supplementary Table S2; Cai et al., 2007; Colombo et al., 2018, 2020), yielding 114 identified *L. casei* strains from 99 samples that were isolated and freeze-dried in a cryoprotectant (Supplementary Table S1, set B).

### Acid production capacity

**Figure 1:**
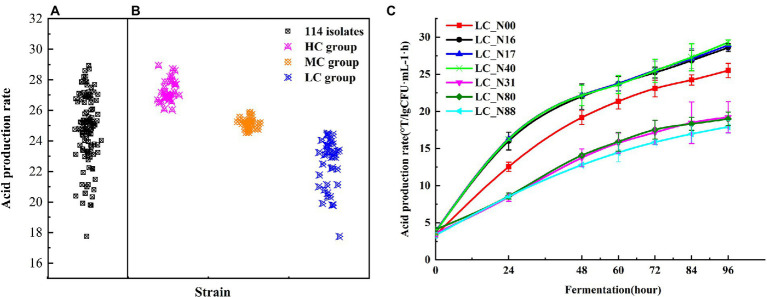
Acid production rate of *L. casei*. **(A)** Acid production rate of 114 isolates fermented for 96 h. **(B)** Acid production rate of HC, MC, and LC strains. **(C)** Acid production rate of representative strains from the HC (LC_N16, 17, 40) and LC (LC_N31, 80, 88) groups, up to 96 h, compared with the commercial strain LC_N00. Tests were performed in triplicate and were expressed as means ± SD. Analysis of variance (ANOVA) was performed by Tukey’s test for pairwise comparison.

All strains successfully induced coagulation of the milk after 114 *l.casei* strains were fermented in milk. The pH of all milk fermentations was reduced below 4.0, microbial counts were greater than 8.0 logCFU g^−1^, and acidity was greater than 160°T for all strains. Of these, 24 strains produced greater acidity than the commercial strain, LC_N00 (238.76 ± 0.46°T; Supplementary Table S3; Figure 1A).

The acid production rate was calculated by dividing the acidity by the viable microbial count. Cluster analysis was used to classify the acid production rate of all *L. casei* strains into three groups, by comparison with the acid production rate of LC_N00. The high acid (HC) group (34 strains) had acid production rates higher than 26.03 ± 0.62, the medium acid (MC) group (38 strains) had acid production rates between 24.54 ± 0.15 and 25.88 ± 0.53 (i.e., similar to that of LC_N00), and the low acid (LC) group (42 strains) had acid production rates less than 24.50 ± 0.25 (Figure 1B). Three exemplar strains were selected from both the HC and LC groups, to compare their acid production rates during fermentation. The mean acid production rates of the HC and LC strains rapidly increased to 22.97 ± 0.97 and 15.38 ± 1.35, respectively, within 60 h, then slowly increased further to 28.68 ± 0.89 and 18.71 ± 0.90, respectively, between 60 h and 96 h (*p* < 0.05; Figure 1C). The mean acid production rates of the HC group were about nine times higher than those of the LC group at 96 h (Table 1).

**Table 1:** Acid production rates of exemplar *L. casei* strains from the high acid (HC) and low acid (LC) groups.

Strain	pH	Titratable acidity (°T)	Viable cell counts (logCFU·g^−1^)	Acid production rate	Group
LC_N17	3.51 ± 0.01^de^	256.83 ± 0.81^b^	8.88 ± 0.04^c^	28.93 ± 0.22^b^	HC
LC_N40	3.50 ± 0.01^de^	256.69 ± 1.37^b^	8.94 ± 0.13^c^	28.70 ± 0.61^b^	HC
LC_N16	3.49 ± 0.01^e^	255.10 ± 1.49^b^	8.92 ± 0.05^c^	28.59 ± 0.17^b^	HC
LC_N00^a^	3.54 ± 0.01^d^	238.76 ± 0.47^c^	9.47 ± 0.02^b^	25.22 ± 0.68^c^	MC
LC_N31	3.81 ± 0.10^c^	177.94 ± 1.97^d^	8.99 ± 0.07^c^	19.81 ± 0.30^d^	LC
LC_N80	3.70 ± 0.11^de^	180.27 ± 3.44^d^	9.21 ± 0.10^b^	19.80 ± 0.16^d^	LC
LC_N88	3.97 ± 0.02^b^	161.24 ± 3.97^e^	8.52 ± 0.08^c^	18.92 ± 0.80^d^	LC

^a^Commercial strain. ^bcde^Different superscript letters indicate significant differences (*p* < 0.05). Tests were performed in triplicate and results are expressed as mean ± standard deviation (SD). Analysis of variance (ANOVA) was performed by Tukey’s range test, for pairwise comparison.

### Location of the *lac* operon

Comparative genomic analysis of the six sequenced HC and LC strains revealed 2,187 core genes (Supplementary Table S4A). The core genes were mapped to the Clusters of Orthologous Groups (COG) database (Huerta-Cepas et al., 2016) to evaluate the main functional COG categories. A collection of abundant COG categories, which contain genes that typically have the same functional category, from the six strains is shown in Supplementary Tables S4B,C. The most common COGs were those associated with “Carbohydrate transport and metabolism” (G). 10.53% (HC) and 9.73% (LC) of the COGs were grouped into the G category (Supplementary Figure S5). Notably, LC_N16 (208), LC_N17 (207), and LC_N40 (207) had almost equal numbers of genes classified into the G category. That is, the homologous gene function annotations indicate that these three strains have similar carbohydrate metabolic pathways.

**Figure 2:**
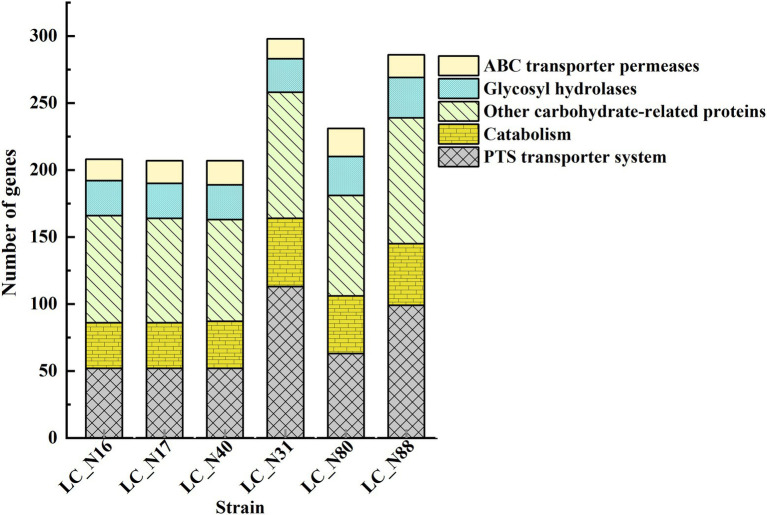
Functional analysis of genes in the exemplar HC and LC strains. Genes related to carbohydrate metabolism. Protein sequences were subjected to an orthology prediction using OrthoMCL with thresholds of: E-Value, 1E-5; percent identity cutoff, 0; and Markov inflation index, 1.5. Core genomes were mapped to the bacterial NCBI COGs and KEGG database to evaluate the main COG categories.

A comparative analysis of metabolic pathways related to genes in the G category indicated that the genes of the HC and LC groups are involved in carbohydrate metabolism, including phosphoenolpyruvate-carbohydrate phosphotransferase (PTS) transporter systems, glycosyl hydrolases, ABC transporter permeases, and other carbohydrate-related proteins (Figure 2). PTS system genes (52 and 91 genes in HC and LC, respectively) were the most numerous. These genes are often arranged in clusters, each of which is involved in the uptake and subsequent hydrolysis of a particular carbohydrate; the ability of strains to metabolize carbohydrates is reflected in their rates of organic acid production and the final concentration achieved (Rasko et al., 2005; Buron-Moles et al., 2019).

**Figure 3:**
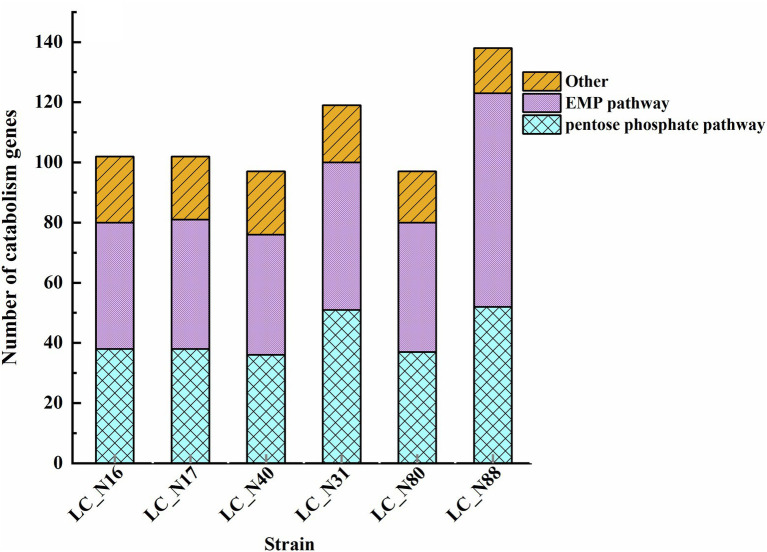
Genes related to lactose catabolism. The genes involved in the hydrolysis of lactose were related to three lactose metabolic pathways: 00052MN-Galactose metabolism (Supplementary Figure S1A), 00010MN-Glycolysis Gluconeogenesis (Supplementary Figure S1B), and 00030 M-Pentose phosphate pathway (Supplementary Figure S1C).

Further analysis was focused on lactose metabolism and revealed that there appear to be two main routes for lactose catabolism in HC and LC strains, namely, the phosphoenolpyruvate-lactose phosphotransferase (PTS^Lac^) system and the lactose permease symport system (Supplementary Table S4). The genes involved in the hydrolysis of lactose are related to three lactose metabolic pathways: 00052MN-Galactose metabolism (Supplementary Figure S1A), 00010MN-Glycolysis Gluconeogenesis (Supplementary Figure S1B), and 00030 M-Pentose phosphate pathway (Supplementary Figure S1C). The main routes of lactose hydrolysis were the Embden–Meyerhof–Parnas (EMP) and pentose phosphate pathways (Figure 3). A comparison of the number, location, and amino acid sequence of the genes in these pathways revealed that the *lac* operon was present in all six strains of the HC and LC groups, but there were significant differences in the location of the *lac* operon encoding PTS^Lac^. The *lacT, E, G,* and *F* genes, which constitute the *lac* operon, control lactose metabolism; *lacT* codes for a transcriptional anti-terminator, *lacE* and *F* for the PTS^Lac^ EIICBA domains, and *lacG* for the phospho-β-galactosidase (Alpert and Siebers, 1997; Zheng et al., 2015). Notably, the *lac* operon responsible for lactose transport and hydrolysis was located on plasmids in the HC strains, whereas that of the LC strains was located in the chromosome (Table 2).

**Table 2:** Location of *lac* operon in exemplar *L. casei* strains from the high acid (HC) and low acid (LC) groups.

Species	Strain	Group	Location	Gene ID	Sequence Type	Gene description
*L. casei*	LC_N16	HC	Scaffold71	gene2587	+	lactose PTS system EIIA component [EC:2.7.1.207]
*L. casei*	LC_N16	HC	Scaffold71	gene2588	+	6-phospho-beta-galactosidase [EC:3.2.1.85]
*L. casei*	LC_N16	HC	Scaffold71	gene2589	+	PTS system lactose-specific EIICB component
*L. casei*	LC_N16	HC	Scaffold71	gene2590	+	Transcription antiterminator LacT
*L. casei*	LC_N17	HC	Scaffold72	gene2592	+	lactose PTS system EIIA component [EC:2.7.1.207]
*L. casei*	LC_N17	HC	Scaffold72	gene2593	+	6-phospho-beta-galactosidase [EC:3.2.1.85]
*L. casei*	LC_N17	HC	Scaffold72	gene2594	+	PTS system lactose-specific EIICB component
*L. casei*	LC_N17	HC	Scaffold72	gene2595	+	Transcription antiterminator LacT
*L. casei*	LC_N40	HC	Scaffold70	gene2578	+	PTS system lactose-specific EIIA component
*L. casei*	LC_N40	HC	Scaffold70	gene2579	+	6-phospho-beta-galactosidase [EC:3.2.1.85]
*L. casei*	LC_N40	HC	Scaffold70	gene2580	+	PTS system lactose-specific EIICB component
*L. casei*	LC_N40	HC	Scaffold70	gene2581	+	Transcription antiterminator LacT
*L. casei*	LC_N31	LC	Scaffold2	gene0480	#	PTS system lactose-specific EIICB component
*L. casei*	LC_N31	LC	Scaffold2	gene0481	#	6-phospho-beta-galactosidase [EC:3.2.1.85]
*L. casei*	LC_N31	LC	Scaffold2	gene0482	#	lactose PTS system EIIA component [EC:2.7.1.207]
*L. casei*	LC_N31	LC	Scaffold2	gene0479	#	Transcription antiterminator LacT
*L. casei*	LC_N80	LC	Scaffold12	gene1054	#	lactose PTS system EIIA component [EC:2.7.1.207]
*L. casei*	LC_N80	LC	Scaffold12	gene1055	#	6-phospho-beta-galactosidase [EC:3.2.1.85]
*L. casei*	LC_N80	LC	Scaffold12	gene1056	#	PTS system lactose-specific EIICB component
*L. casei*	LC_N80	LC	Scaffold12	gene1057	#	Transcription antiterminator LacT
*L. casei*	LC_N88	LC	Scaffold5	gene1060	#	PTS system lactose-specific EIICB component
*L. casei*	LC_N88	LC	Scaffold5	gene1061	#	6-phospho-beta-galactosidase [EC:3.2.1.85]
*L. casei*	LC_N88	LC	Scaffold5	gene1062	#	lactose PTS system EIIA component [EC:2.7.1.207]
*L. casei*	LC_N88	LC	Scaffold5	gene1059	#	Transcription antiterminator LacT

+Located on plasmid and #Located on chromosome.

### Lactose assimilation

**Figure 4:**
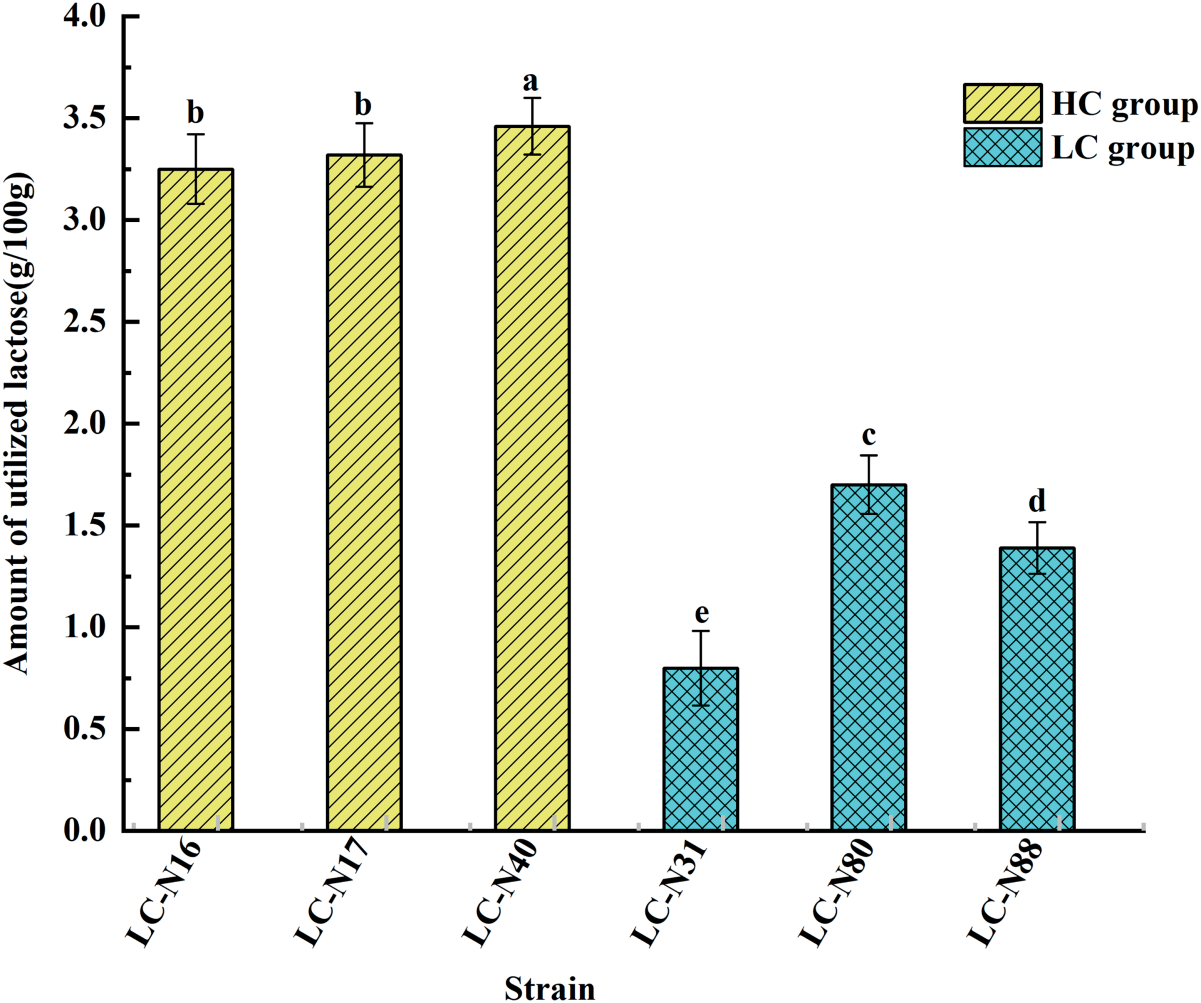
Lactose utilization by the six exemplar HC and LC strains. Relative amounts of lactose utilized by the exemplar HC and LC strains. The analyses were performed in triplicate and the results expressed as mean ± SD. ^abcde^Different superscript letters indicate significant differences (*p* < 0.05).

The lactose catabolic capacity of the six HC and LC group exemplar strains was determined by measuring the lactose content of hydrolyzed milk by anion-exchange high-performance liquid chromatography (HPLC; Richmond et al., 1982; Zhang et al., 2010). The linearity of the chromatographic method was verified by the coefficient of determination (𝑅^2^), which was >0.99 and the retention time of lactose was 19.39 ± 0.12 min, which indicates good reproducibility (Supplementary Figure S6). In addition, all six strains reached a turbidity in MRS medium of OD_600_ > 0.8 and a pH < 5.0 within 24 h, indicating that lactose was catabolized to produce organic acids (results not shown). The lactose utilization rates of the HC strains (LC_N16, 17, 40) were 46.4, 47.5, and 49.4%, respectively, whereas those of the LC strains (LC_N31, 80, 88) were 11.4, 24.3, and 19.8%, respectively, so the lactose utilization rate of HC strains was markedly higher than that of LC strains (Figure 4; *p* < 0.05). The *lac* operons of HC strains were located on plasmids and it appears that this enabled higher expression of the genes responsible for lactose metabolism, enabling these strains to rapidly catabolize lactose into glucose and galactose-6 phosphate.

### Copy numbers of *lac* operon

Comparison of copy numbers of *lac* operon in HC and LC strains. The *lac* operon copy number in the six exemplar strains was determined by real-time PCR. A standard curve was generated for the *lacG* and *tuf* genes by linear regression of a plot of serial five-fold dilutions of both plasmid and genomic DNA (Table 3). Theoretically, the slope of the standard curve should be computed as its absolute gradient (−1/log_5_2 = −2.32; Lee et al., 2006); the standard curves obtained for the *lacG* and *tuf* genes were linear over the tested range (𝑅^2^ > 0.99) and the slopes were between −2.23 and −2.41, only slightly different (<2%) from the theoretical value (Table 3). The copy numbers of the HC strains (LC_N16, 17, 40) were 9.34 ± 2.09, 3.09 ± 1.02, and 6.39 ± 0.98, respectively, significantly higher than those of the LC strains (LC_N31, 80, 88), at 1.2 ± 0.16, 1.14 ± 0.12, and 1.16 ± 0.10, respectively (Table 3). Plasmids are genetic material independent of the chromosome, which can replicate autonomously and their genes can be expressed to much higher levels than chromosomal genes (Panya et al., 2012). High copy numbers of *lac* operon genes would facilitate HC strains to catabolize lactose more rapidly than LC strains, in agreement with the lactose utilization results (Figure 4).

**Table 3:** Copy numbers of *lac* operon in exemplar *L. casei* strains from the high acid (HC) strains (LC_N16, 17, 40), recombinant strains (LC_N31-, 80-, 88–601), control strains (LC_N31-, 80-, 88–600) and LC wild-type strains (LC_N31, 80, 88).

Strain	Gene	Amplification efficiency	Average ΔC_T_^a^	Copy number
LC_N16	*lacG*	103.84%	−3.20 ± 0.32	9 ± 2^d^
	*tuf*	103.84%		
LC_N17	*lacG*	103.84%	−1.56 ± 0.48	3 ± 1 ^f^
	*tuf*	98.35%		
LC_N40	*lacG*	97.78%	−2.66 ± 0.22	6 ± 1^e^
	*tuf*	97.78%		
LC_N31	*lacG*	97.78%	−0.25 ± 0.09	1 ± 1^g^
	*tuf*	104.48%		
LC_N80	*lacG*	104.48%	−0.19 ± 0.15	1 ± 1^g^
	*tuf*	98.35%		
LC_N88	*lacG*	103.84%	−0.21 ± 0.12	1 ± 1 ^g^
	*tuf*	98.93%		
LC_N31-600	*lacG*	99.52%	−0.01 ± 0.02	1 ± 1^g^
	*tuf*	101.94%		
LC_N31-601	*lacG*	103.20%	−3.98 ± 0.13	16 ± 1^c^
	*tuf*	101.94%		
LC_N80-600	*lacG*	100.60%	−0.15 ± 0.03	1 ± 1 ^g^
	*tuf*	101.94%		
LC_N80-601	*lacG*	99.52%	−4.05 ± 0.03	17 ± 1^bc^
	*tuf*	99.52%		
LC_N88-600	*lacG*	103.20%	−0.30 ± 0.13	1 ± 1^g^
	*tuf*	99.52%		
LC_N88-601	*lacG*	103.20%	−4.17 ± 0.15	18 ± 2^b^
	*tuf*	100.71%		

^a^ΔC_T_, the C_T_ deviation of *lacG* vs. *tuf* was calculated for each dilution (*n* = 3). ^bcdefg^Different superscript letters indicate significant differences (*p* < 0.05). Average ΔC_T_ = means ± SD (*n* = 5). Serial five-fold dilutions of both plasmid and genomic DNA were conducted to establish the standard curves. The standard curve was a plot of the threshold cycle (C_T_) versus log concentration (Co); by interpolating their C_T_ values against the standard curve, the absolute quantity of both plasmid and genomic DNA was obtained.

Copy numbers of *lac* operon in recombinant strains. To determine the relationship between copy number and acid production rate, the *lacT, E, G, and F* genes were cloned into the pSIP600 vector (Supplementary Figure S3), then transferred into the LC strains by electroporation (Welker et al., 2015). The recombinant vector was verified by PCR (lacT-lacE-F/R and lacG2-F/R) and digested with *Hind*III, to confirm successful construction of pSIP601 (Supplementary Figure S4). The copy numbers of *lac* operon genes in the recombinant strains LC_N31-601, LC_N80-601, and LC_N88-601 were 15.86 ± 1.34, 16.59 ± 0.38, and 18.12 ± 1.90, respectively, and were significantly higher than those of the control (LC_N31-600, LC_N80-600, LC_N88-600) and LC wild-type strains (LC_N31, LC_N80, LC_N88; Table 3; *p* < 0.05). The pSIP series vectors are one-plasmid systems with pUC(pGEM)ori-256rep replication determinants for *Escherichia coli*, *Lactobacillus sakei*, and *Lactiplantibacillus plantarum*; the replication determinants of pSIP600 vector can be changed easily, meaning that the system can be made to function well in Lactobacilli (Sorvig et al., 2003). Markedly increasing the copy numbers of *lac* operon genes in the recombinant strains should increase their expression.

### Performance of recombinant strains

**Figure 5:**
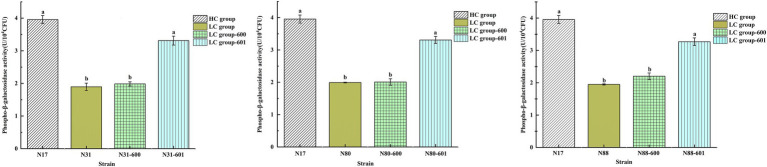
Activity of LacG (phospho-β-galactosidase) in *L. casei* strains. Assays were performed in triplicate and the results are presented as mean ± SD. ^ab^Different superscript letters indicate significant differences (*p* < 0.05).

Phospho-β-galactosidase activity of recombinant strains. Phospho-β-galactosidase (LacG) is involved in the catabolism of lactose and is required for lactose utilization by LAB. LacG activity was assayed with *o*-nitrophenyl-β-D-galactopyranoside-6-phosphate (ONPG-6-P). The LacG activities of the recombinant strains (LC_N31-601, LC_N80-601, LC_N88-601) were 3.31 ± 0.14, 3.31 ± 0.11, and 3.27 ± 0.12 U/10^4^ CFU, respectively (Figure 5) and were not significantly different from the HC strains (*p* < 0.05), but were markedly higher than the LC-600 control and LC wild-type strains. These results suggested that the high copy numbers of the *lacG* gene in the recombinant LC strains increased both *lacG* expression and their LacG activity, which would increase their hydrolytic capacity for intracellular phosphorylated lactose.

**Figure 6:**
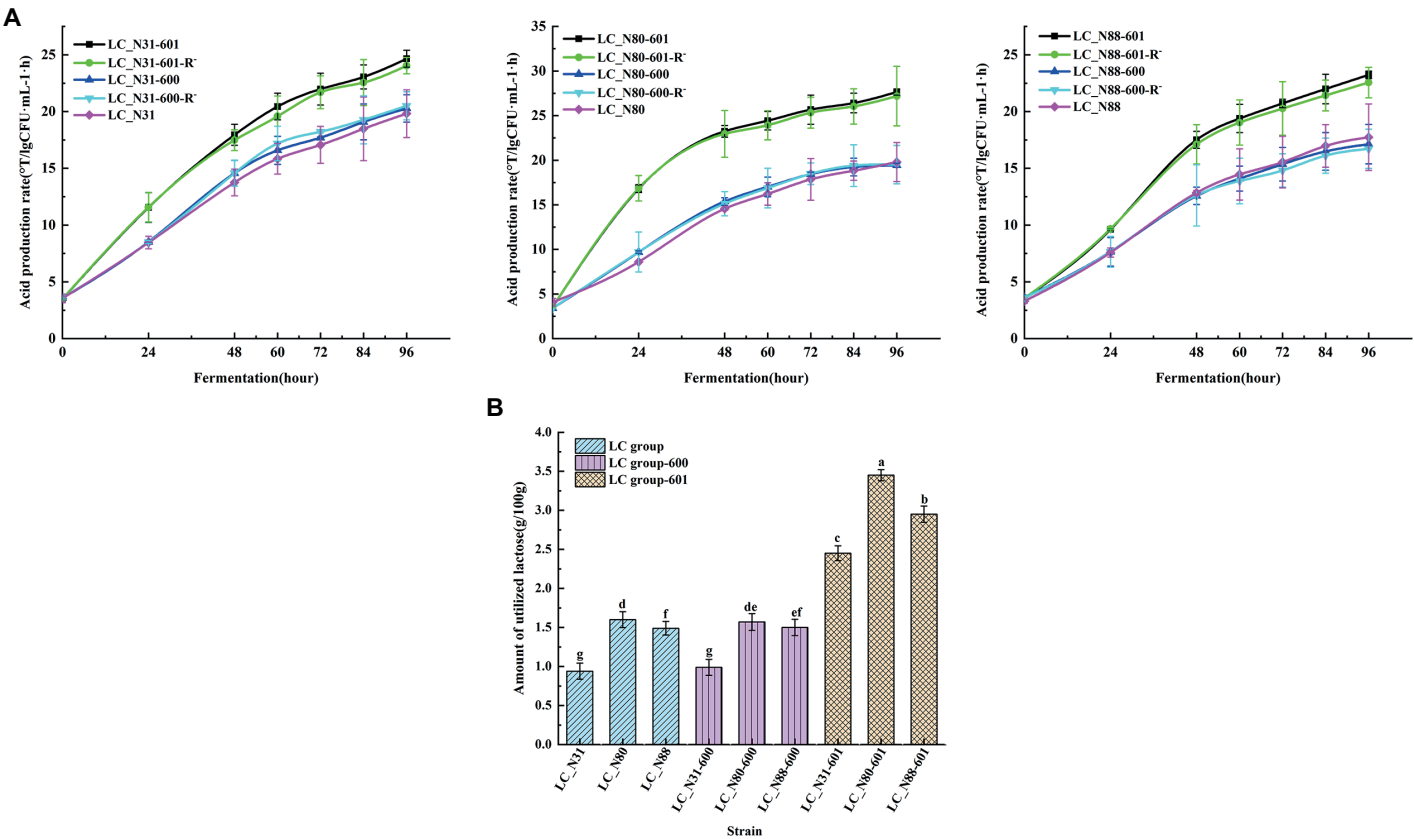
**(A)** Acid production rate of LC recombinant and wild-type strains. **(B)** Lactose utilization by recombinant and wild-type strains. Assays were performed in triplicate and are expressed as mean ± SD. ^abcdefg^Different superscript letters indicate significant differences (*p* < 0.05).

Performance of recombinant strains in milk fermentation. The acid production rates of the recombinant LC strains in milk fermentations, with and without erythromycin resistance, were compared with those of the corresponding wild-type strains. There was no significant difference between the recombinant LC strains with erythromycin resistance (LC_N31-601, LC_N80-601, LC_N88-601) and without (LC_N31-601-R^−^, LC_N80-601-R^−^, LC_N88-601-R^−^), indicating that the *lac* operon is stable in the recombinant strains (Figure 6A; *p* < 0.05). However, the acid production rates of the recombinant LC strains were significantly higher than the recombinant control and LC wild-type strains (Figure 6A; *p* < 0.05). The lactose utilization of the recombinant LC strains (35.0, 50.7, and 42.1%) was significantly higher than LC wild-type (13.1, 21.5, and 17.9%) and control strains (14.2, 22.9, and 21.4%; Figure 6B; *p* < 0.05). These results suggest that location of the *lac* operon on a plasmid in *L. casei* markedly accelerates lactose utilization and increases organic acid production during milk fermentation.

## Discussion

The acid production rate of *L. casei* is an important characteristic, which determines the quality of fermented milk beverages and influences the cost of industrial milk fermentation. Some measures of acid production rate have been reported, the pH, t_Vmax_, t_pH4.5_, and the kinetic parameter Vm = dpH/dt were quantified to assess *Streptococcus* strains (Zanatta and Basso, 1992; Dashper et al., 2012) and *Lactobacillus acidophilus* (Almeida et al., 2008). Fugl et al. (2017) evaluated the fermentation performance of *L. lactis*, *S. lutetiensis*, *S. infantarius*, and *P. acidilactici* strains by acidity and pH. These parameters take into account one important indicator of the starter, but the final microbial count in the fermented probiotic product must also be considered. Therefore, this study used acid production rate to assess LAB strains, which can be calculated from the acidity and viable cell count. The resulting acid production rate data were used to classify the *L. casei* isolates into three groups: the “medium acid” (MC) strains had similar acid production capacity to the commercial starter strain LC_N00, which has industrially acceptable acid production and final microbial counts. The “high acid” (HC) and “low acid” (LC) strains had higher and lower acid production capacity, respectively, in milk fermentations, compared with LC_N00. The HC and MC strains not only met industrial acid production requirements, but also could reach a final viable count of >10^8^ CFU/ml.

Uptake of lactose into bacterial cells and initiation of its catabolism involves several metabolic pathways: ABC protein-dependent systems, lactose-galactose antiporters, lactose-H^+^ symport systems, and the PTS^Lac^ transporter system (Alpert and Siebers, 1997). The two main systems active in the exemplar *L. casei* strains studied here were the lactose permease symport and PTS^Lac^ systems. Although there are at least two sugar transport systems, most sugars are transported by PTSs, the primary sugar transport systems of Gram-positive bacteria (Ajdic et al., 2002). PTSs regulate overall carbohydrate metabolism through gene expression in *L. casei*, and are important under acidic conditions (Wu et al., 2012).

Lactose fermentation is very widely employed and well-understood by the dairy industry (Cavanagh et al., 2015). LAB have evolved metabolic systems that ensure the preferential use of readily metabolizable carbon sources, such as carbon catabolite repression (CCR), which modulates gene expression in response to the availability of carbon compounds; CCR is well known in lactobacilli and can also interact with catabolite control protein A (CcpA), the master regulator of carbon metabolism (Monedero et al., 1997; Stefanovic et al., 2017). Inhibition of *lac* gene expression during growth on glucose is a consequence of PTS^Glc^-mediated inducer exclusion, a repressive effect of a functional glucose-phosphoenolpyruvate-dependent phosphotransferase system (Chassy and Thompson, 1983; Monedero et al., 1997). However, the *L. casei* strains studied here were able to utilize three sugars (lactose, sucrose, and glucose) simultaneously, but with lactose as the primary carbon source (HC and LC strains utilized mostly lactose; results not shown). During glucose fermentation by *L. casei via* the EMP, carbohydrates are preferentially transported by PTS systems, and metabolism of disaccharides is preferred over glucose fermentation *via* the pentose phosphate pathway (Ganzle et al., 2007; Ganzle and Follador, 2012). When combined, these two metabolic shifts increase the yield of ATP. Accordingly, metabolism of lactose and sucrose by disaccharide phosphorylases is not repressed by glucose.

The *lac* operon genes *lacT, E, G, and F*, which constitute PTS^Lac^, regulate lactose metabolism during milk fermentation (Bidart et al., 2018). The *lac* operon is induced by lactose through transcription antitermination, mediated by *LacT* (Gosalbes et al., 1999) and several *L. casei* strains (e.g., *L. casei* 334/64H) carry the *lac* operon on a plasmid (Gosalbes et al., 1997; Van Kranenburg et al., 2005). Unique functions of *L. casei* are encoded on mobile elements, such as plasmids or transposons, including exopolysaccharide biosynthesis and sugar metabolism; these functions contribute to the high viscosity, i.e., creamy texture of fermented milk beverages and to utilization of lactose in dairy fermentations (Davidson et al., 1996; Schroeter and Klaenhammer, 2009).

Differences in *L. casei* lactose metabolism resulting from different locations of the *lac* operon (plasmid vs. chromosome) have not been previously reported. This is the first report that expression of the *lacT, E, G, and F* genes located on a plasmid is enhanced, compared with the same genes located on the chromosome, since the former can replicate autonomously. The high expression of the *lacG* gene increased LacG enzyme activity and consequently, lactose hydrolysis, which increased the acid production rate, an important attribute of a milk fermentation starter culture.

In conclusion, the association between acid production rate and lactose metabolism enabled the identification of the genes involved in transport and catabolism of lactose. Further research is required to confirm these findings, as well as to elucidate the potential functions of these genes during milk fermentation, which is of great importance for industrial applications of *L. casei*.

## Data availability statement

The datasets presented in this study can be found in online repositories. The names of the repository/repositories and accession number(s) can be found in the article/Supplementary material.

## Author contributions

XL: conceptualization, data curation, methodology, software, and writing—original draft. ZZhai and YH: formal analysis, writing—review and editing, and resources. MZ and CH: software and supervision. JH, SS, ZZhao, and YS: validation and visualization. FR and RW: conceptualization, data curation, formal analysis, investigation, project administration, resources, and supervision. All authors contributed to the article and approved the submitted version.

## Funding

This work was supported by the National Natural Science Foundation of China (contract 31972055) and the National Key Research and Development Program of China (2018YFC1604303).

## Conflict of interest

The authors declare that the research was conducted in the absence of any commercial or financial relationships that could be construed as a potential conflict of interest.

## Publisher’s note

All claims expressed in this article are solely those of the authors and do not necessarily represent those of their affiliated organizations, or those of the publisher, the editors and the reviewers. Any product that may be evaluated in this article, or claim that may be made by its manufacturer, is not guaranteed or endorsed by the publisher.
