# Supplementary figures and images for: The plasmid-encoded lactose operon plays a vital role in the acid production rate of Lacticaseibacillus casei during milk beverage fermentation
Source: Front Microbiol. 2022 Oct 6;13:1016904. doi: 10.3389/fmicb.2022.1016904 (PMC9647812; doi:10.3389/fmicb.2022.1016904)

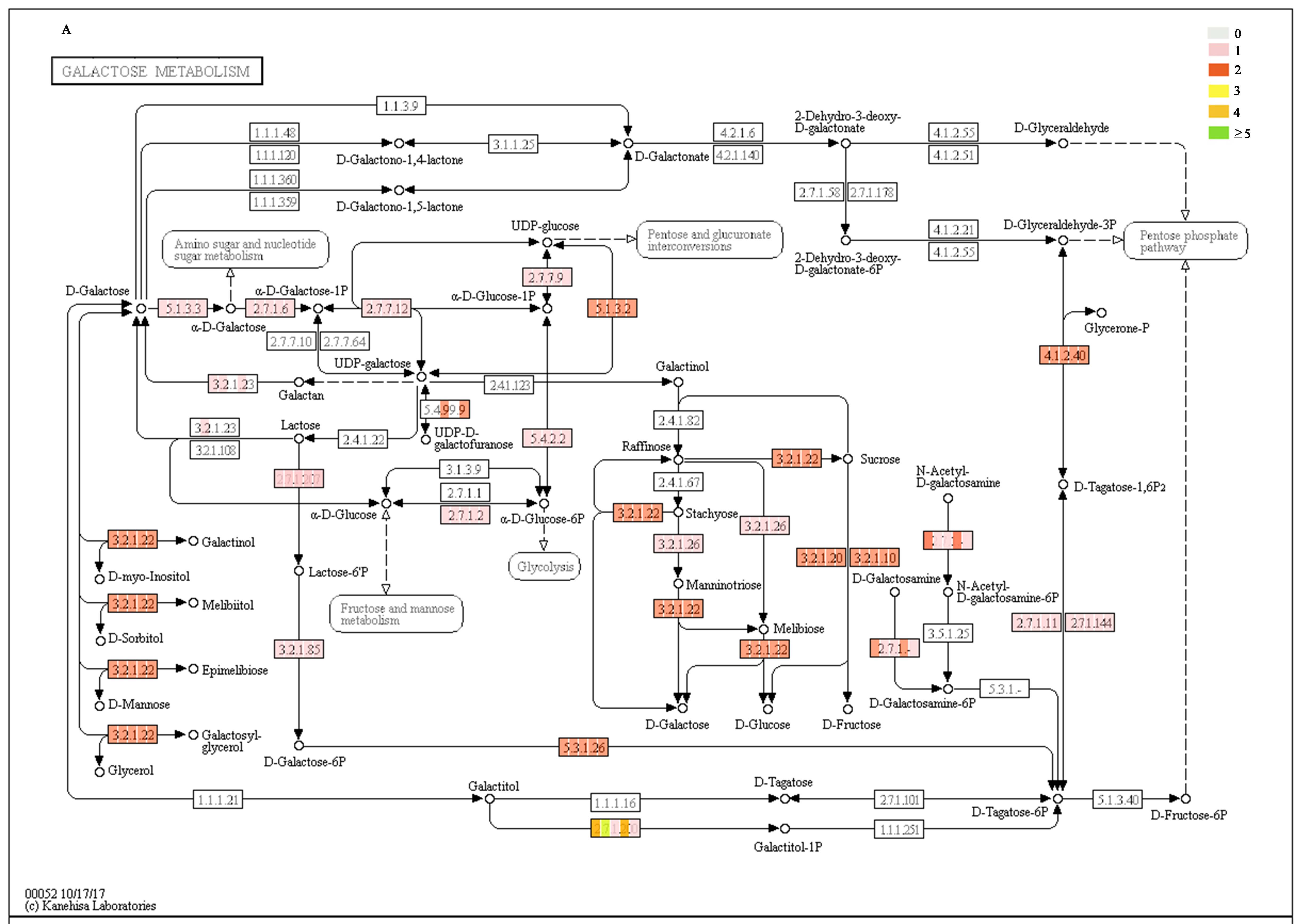

Supplement: Supplementary file 5 [file Image_1.TIF]

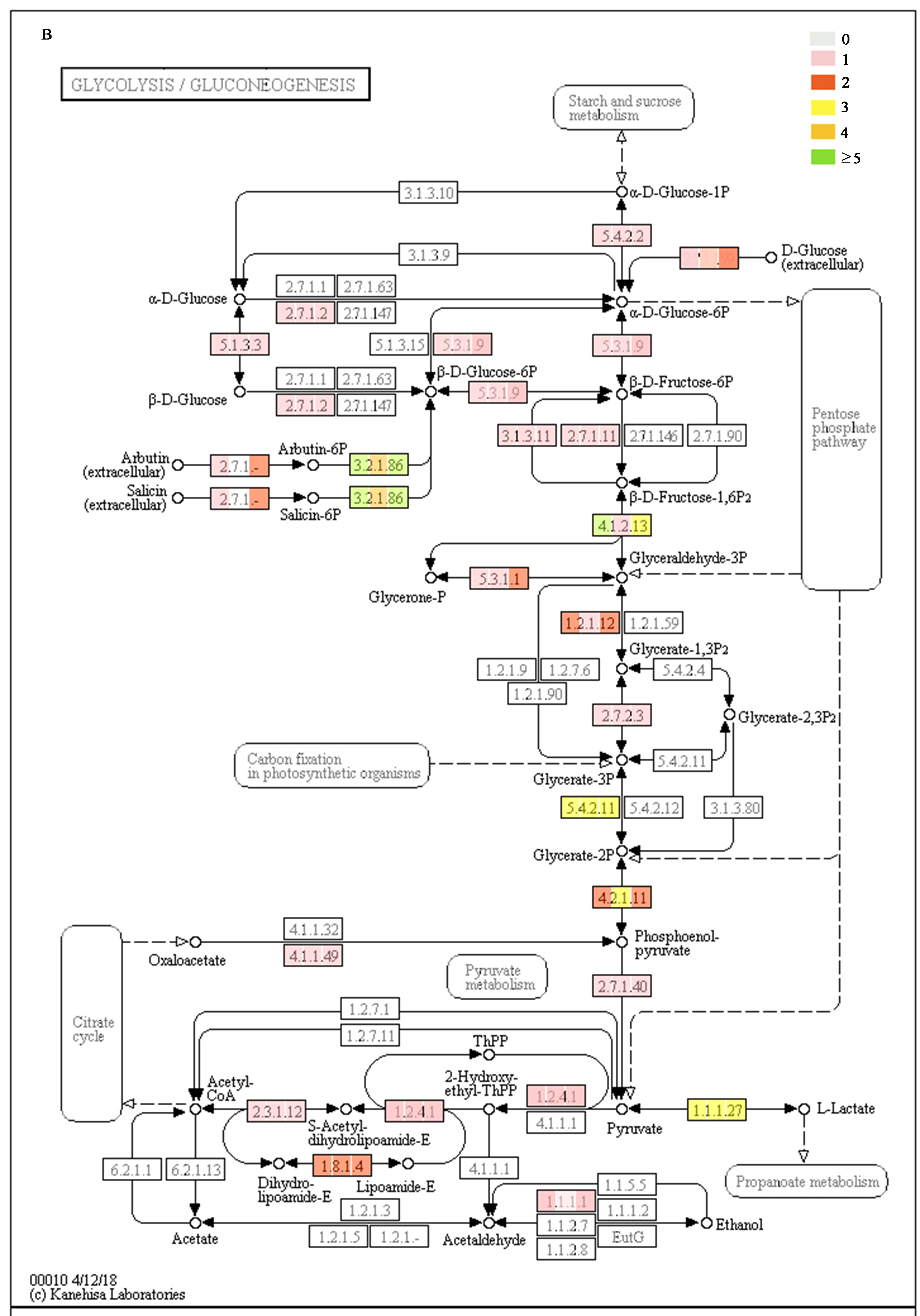

Supplement: Supplementary file 6 [file Image_2.TIF]

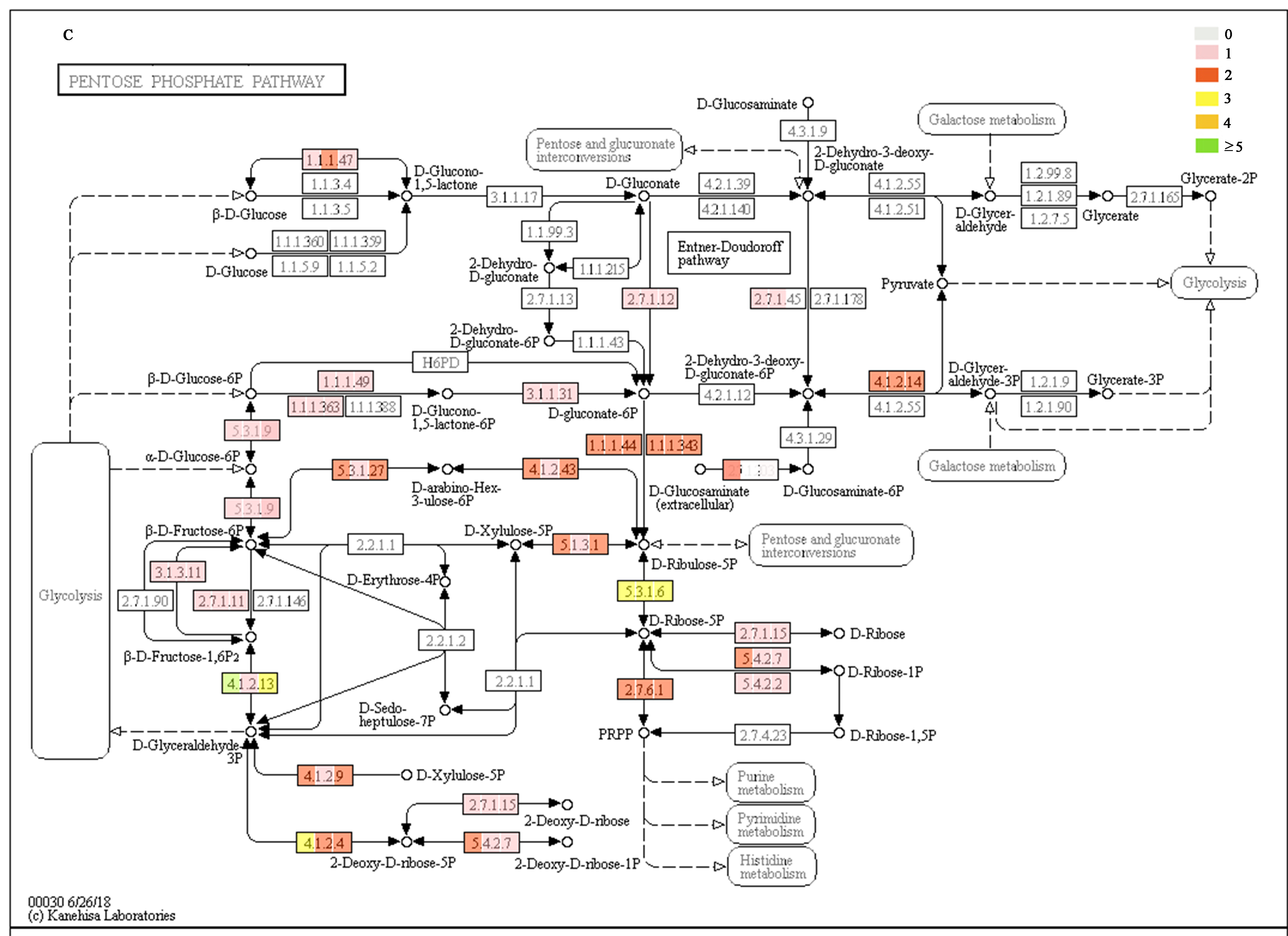

Supplement: Supplementary file 7 [file Image_3.TIF]

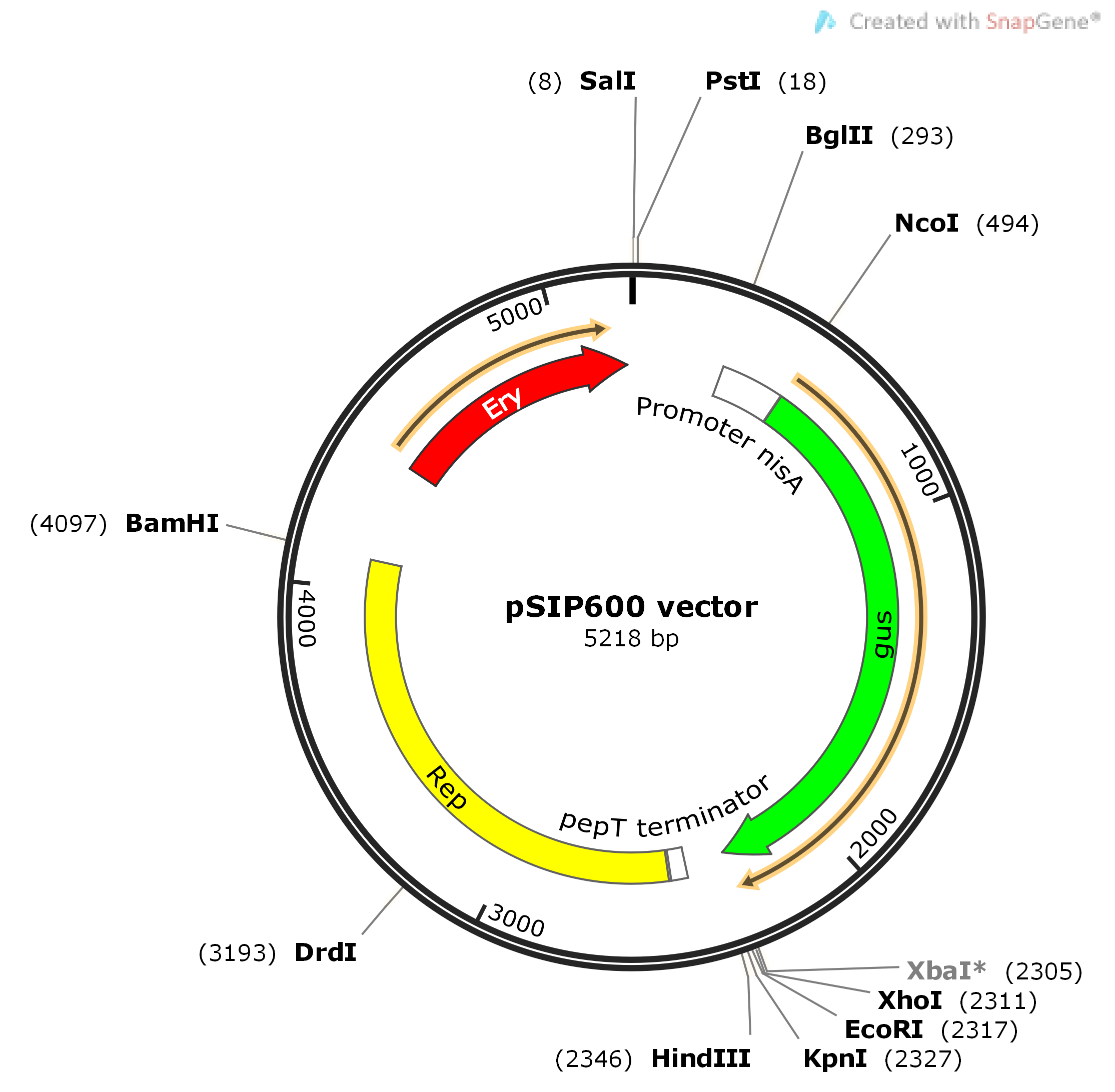

Supplement: Supplementary file 8 [file Image_4.TIFF]

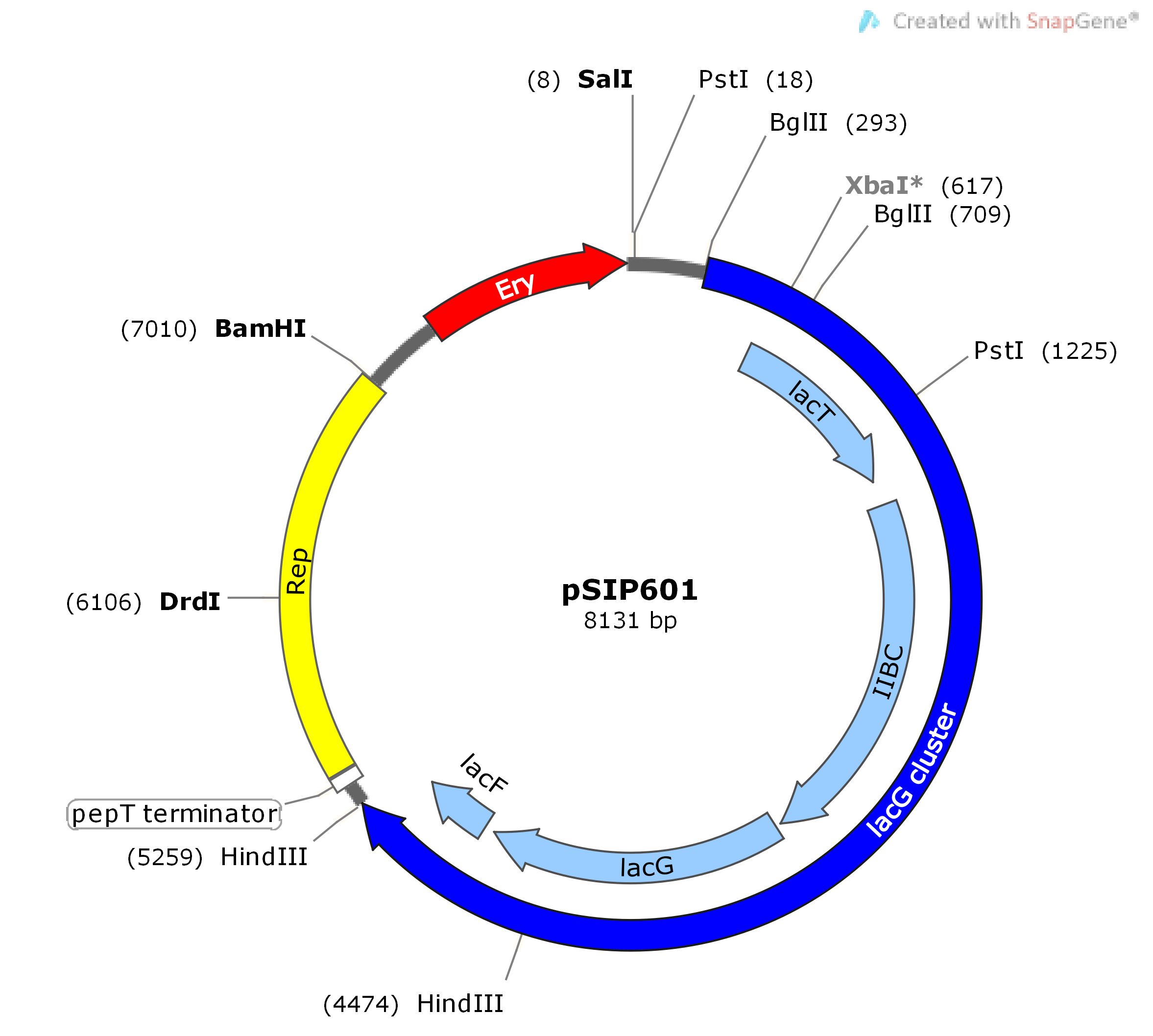

Supplement: Supplementary file 9 [file Image_5.TIFF]

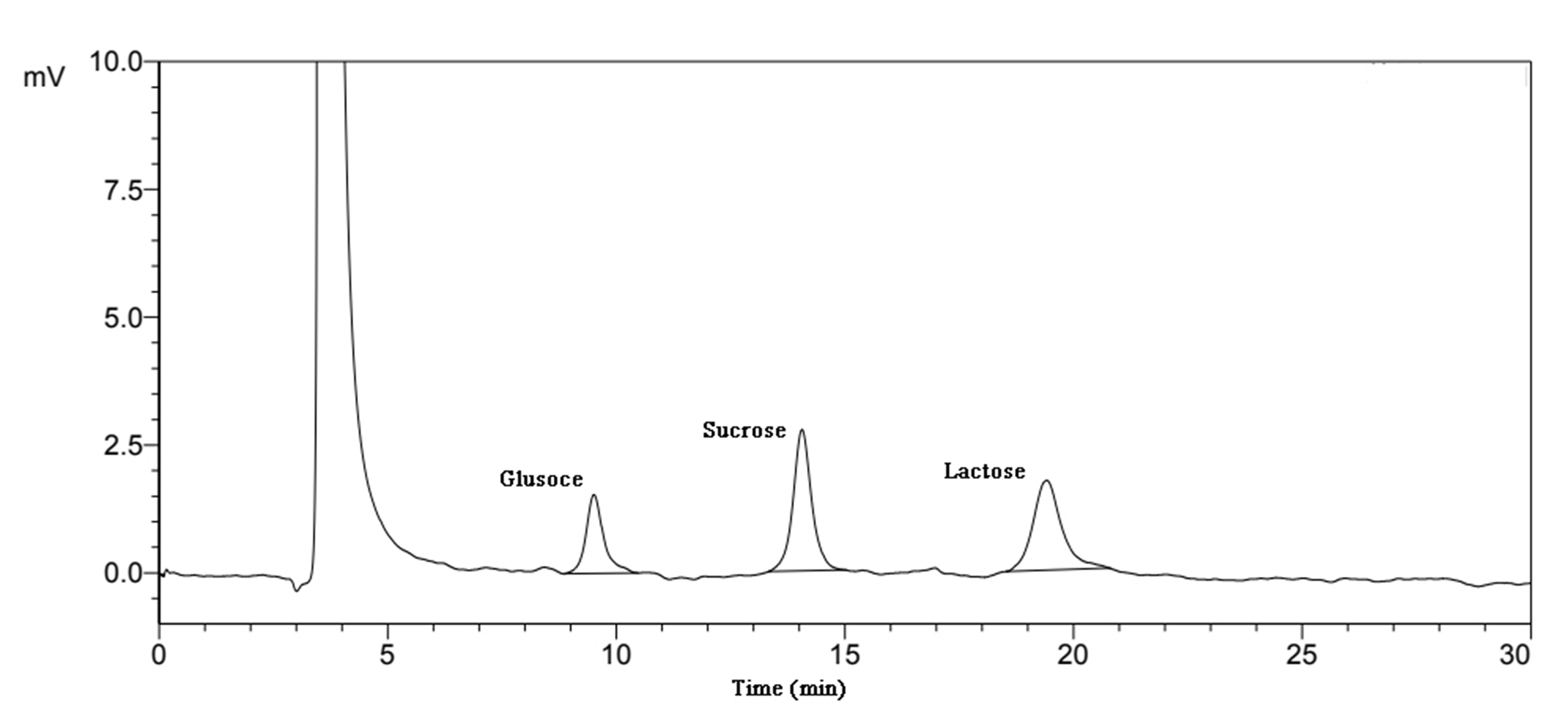

Supplement: Supplementary file 12 [file Image_8.TIF]
